# Nursing Patient Classification Systems to Assess Pediatric Patients’ Nursing Complexity: An Empty Narrative Literature Review

**DOI:** 10.3390/healthcare13222923

**Published:** 2025-11-15

**Authors:** Roberta Da Rin Della Mora, Silvia Rossi, Ilaria Artuso, Simona Calza, Roberta Tirone, Stefano Parodi, Giulia Ottonello, Nicoletta Dasso, Silvia Scelsi

**Affiliations:** 1 Center for Research in Nursing and Health Professions, Health Professions Direction, IRCCS Istituto Giannina Gaslini, 16147 Genova, Italy; robertadarindellamora@gaslini.org; 2 Health Profession Direction, IRCCS Istituto Giannina Gaslini, 16147 Genova, Italy; silviarossi@gaslini.org (S.R.); ilariaartuso@gaslini.org (I.A.); simonacalza@gaslini.org (S.C.); robertatirone@gaslini.org (R.T.); giuliaottonello@gaslini.org (G.O.); silviascelsi@gaslini.org (S.S.); 3 Epidemiology and Biostatistics Unit, Scientific Directorate, IRCCS Istituto Giannina Gaslini, 16147 Genova, Italy; stefanoparodi@gaslini.org

**Keywords:** hospital organization, narrative review, nursing complexity, Nursing Patient Classification System, patient complexity, pediatric patient

## Abstract

**Background/Objectives:** In recent years, some hospitals have shifted from traditional to intensity-of-care-based organizational models, where patients allocation is based on the care they require. In this context, Nursing Patient Classification Systems (NPCSs) can lead to the identification of the appropriate nursing care setting based on patient nursing needs; this also applies to pediatrics. In this scenario, the concept of patient nursing complexity has emerged. This narrative literature review aims to provide an overview on validated NPCSs that assess the nursing complexity of pediatric patients. **Methods:** PubMed, CINAHL, and the Cochrane Library were searched, and inclusion and exclusion criteria were applied to the retrieved papers. Two authors independently screened n = 498 papers, of which n = 7 were read in full and subsequently excluded. **Results:** No paper met the inclusion criteria. However, papers read in full were analyzed, and their main characteristics were described. They were excluded because they did not concern validated NPCSs that assess pediatric patients’ nursing complexity or did not assess pediatric patients’ nursing complexity as defined in this literature review. **Conclusions:** This narrative literature review highlighted a critical gap in the field of validated NPCSs that assess the nursing complexity of pediatric patients. The lack of a shared definition of “nursing complexity of the patient” is the primary barrier identified. This constitutes a crucial take-home message. Therefore, future studies should prioritize in-depth exploration of the differences among all the published definitions and concepts related to “complexity” and harmonizing existing conceptual analysis to guide future research and the development of shared NPCSs.

## 1. Introduction

In recent years, some Italian hospitals have shifted from traditional to intensity-of-care-based organizations. The main difference between those two paradigms is that in the traditional hospital organization, the care delivery system is focused on the disease characteristics, and patients are allocated to wards organized by medical specialty [1]; the intensity-of-care-based hospital, instead, focuses on the patient characteristics, so patients are allocated to the proper setting based on the care they require to satisfy their health-related needs [2].

In this scenario, the importance of the concept of care complexity was highlighted [3].

Since the early 1960s, Patient Classification Systems (PCSs) have been common methods in hospital settings to assess and categorize patients [4]. They have been employed to fulfill patients’ needs of cure and care, evaluate the economic and staffing burden, properly allocate economic resources, and improve working conditions of the healthcare providers [4].

When discussing concepts related to nursing care, PCSs are typically referred to as “Nursing Patient Classification Systems” (NPCSs) [5].

NPCSs enable a suitable assessment of the demand for nursing care required by patients, categorizing them based on their nursing needs [6]. In many of these systems, nursing care categories were defined for the classification of adult patients, making them not always suitable or applicable to children and young people [4].

The patient- and family-centered care approach is strongly emphasized in the pediatric setting; therefore, care for children is not only exclusively focused on the patients but also on their families [7,8].

In the scientific literature, several NPCSs exist, and they are also named instruments or tools aimed at classifying patients. They are based on different concepts: “patient acuity” [9], “patient dependency” [10], “patient complexity”, and “nursing intensity” [11]; “complexity of care” [1,3]; or “complexity in nursing care” or “nursing complexity” [12]. Among the several terms used in the literature, it is not easy to find an agreement between the underlying concepts and their definition, if any. This lack of homogeneity in the use of terms and concepts, without clarity on the nuances of meaning, hinders a clear overview of those NPCSs.

For our purpose, we sought NPCSs aimed at assessing the pediatric patients’ nursing complexity, that is, the complexity of their nursing needs and of nursing care they require, to allocate them to the appropriate setting of nursing care.

This topic has been particularly studied in Italy, but it was performed without a univocal definition of the concept of “complexity” (Table 1).

A concept analysis on the “complexity of care” confirmed the variability in the definition of the concept in the literature, highlighting that the primary use of “complexity” referred to the “quantitative measurement of contextual elements (…) as well as organizational variables.” The results, however, underpinned the idea that, given its capacity to express the essential features of the “qualitative and non-linear characteristics of phenomena”, this concept should be developed and applied in other ways, with a primary focus “on the subjects of care and their classification as a necessary and logically antecedent passage of the strategic definition of hospital reorganization tools” [1].

Later, based on the results of a qualitative study aimed at describing the meaning of “care complexity” from the perspective of nurses [3], the same authors concluded that this concept, for its characteristics, is not a measurable concept, whereas “patient care complexity” is measurable and classifiable.

The lack of homogeneity in definitions can be explained by the assumption that concepts, which are influenced by multiple factors, are in a state of constant development and require ongoing redefinition in a cyclical process [14]. On the other hand, the difficulty in clearly defining a concept can significantly impact its potential to form a foundation for practice [14]. Given the lack of a shared definition in the literature and committed to the assumption that patients’ nursing complexity stems from the complexity of their nursing needs, requiring interventions at different levels of complexity, for our literature review, we defined the patient’s nursing complexity according to the view of Richards and Borgling [15] (p. 531), which was later cited by Sasso et al. [16] as “the quintessential ‘complex intervention’—defined as an activity that contains a number of component parts with the potential for interactions between them which, when applied to the intended target population, produces a range of possible and variable outcomes”.

Currently, such a broad definition (not directly linked to nursing dimensions; not strictly related to patient acuity, patient dependency, or nursing intensity as described in the literature; and independent from organizational issues) could better capture the essence of the nursing complexity of the patients rather than that of their clinical condition or the nurses’ work.

Typically, for assessment tools, classification systems have to undergo validation procedures to provide reliable and valid results [17].

As our pediatric hospital undergoes the change from traditional to intensity-of-care-based organization, a group of nurse scientists was asked by the Health Profession Direction to identify a system for assessing patients’ nursing complexity, leading to their allocation to the appropriate setting of nursing care. Consequently, this narrative literature review aimed to provide an overview on validated Nursing Patient Classification Systems that assess the nursing complexity of pediatric patients as defined above.

## 2. Materials and Methods

### 2.1. Study Design

We adopted a narrative review approach to gain a comprehensive understanding of the topic [18], following, as recommended, a systematic method [19] to define the area of interest, the aim, and the search strategy (the research question, the inclusion and exclusion criteria, and the search queries) to identify, retrieve, and select relevant papers.

### 2.2. Literature Search

**Figure 1:**
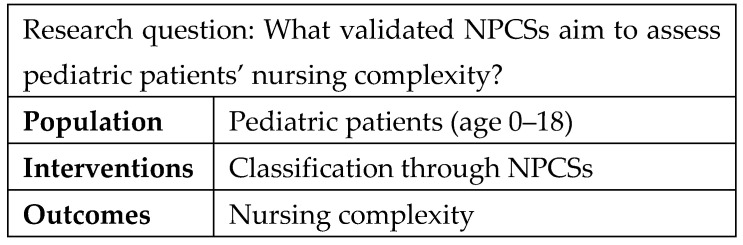
Research question and definition of the area of interest.

The research question was as follows: “What validated Nursing Patient Classification Systems aim to assess pediatric patients’ nursing complexity?”. We then defined the area of interest using the PIO format (Population, Interventions, and Outcomes) [20] (Figure 1). The literature search was conducted at an international level.

Subsequently, we transformed these terms into search terms according to the Medical Subject Headings (MeSH^®^) thesaurus when available. For terms not found in the MeSH^®^ thesaurus, we used nursing-specific terminology verified by the online Cambridge Dictionary (https://dictionary.cambridge.org). The Boolean operator “OR” was used in the search queries to broaden the scope of the search, such as brackets, asterisks, etc.

We searched the PubMed and Cumulative Index to Nursing and Allied Health Literature (CINAHL) databases, as well as the Cochrane Library, using a search query that precisely matched the search terms resulting from the application of the PIO format.

The age limits were set (0–18 years), and English, French, Italian, and Portuguese were included as languages understood by the authors. No date limits were set to enhance the chances of finding articles of interest.

The inclusion and exclusion criteria were defined according to the review’s aim. For a paper to be included in the review, it had to meet the following inclusion criteria: (a) the term “complexity” was present in the title or the abstract; (b) it concerned validated NPCSs aimed at assessing pediatric patients’ nursing complexity as defined in this literature review; (c) it focused on pediatric patients (aged 0–18 years); and (d) it had to be published in English, French, Italian, or Portuguese. Exclusion criteria were as follows: (a) papers concerning not validated NPCSs aimed at assessing pediatric patients’ nursing complexity as defined in this literature review and (b) papers whose full text was not available.

This first query launched on the databases yielded eight articles, none of which were eligible. Therefore, additional queries (recombining the search terms differently) were created and launched on the same databases to yield more results.

The term “complexity” was present in the first research query. However, given the preliminary literature review, which indicates that the term “complexity” has been used to describe different meanings, we decided to employ a broader query by removing it as a search term.

To further broaden the number of retrieved articles, we also removed the search term “pediatr*”, as the pediatric age limits were applied via the database’s filter.

**Figure 2:**
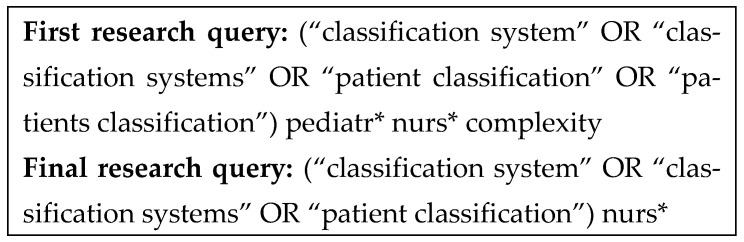
Detailed structure of the research queries on the basis of the areas of interest.

So, the final query for the three databases was as follows: (“classification system” OR “classification systems” OR “patient classification”) nurs* (Figure 2).

Two authors (R.D.R.D.M. and N.D.) independently read the results’ titles and abstracts to assess if they met the inclusion and exclusion criteria and if they were relevant to this study. If there were any doubts, the papers were included in the list of papers to be read in full. Then, they removed duplicates from the list and retrieved and read the full text of the remaining papers to confirm if they were relevant to the study. Finally, they searched the reference list of the papers whose full texts were read and searched the databases again, using the first author’s names of the retrieved relevant papers as search terms, to find other relevant papers.

## 3. Results

The search resulted in a total of n = 496 papers identified from databases; later in the process, two papers were identified by hand-searching the reference lists of relevant papers. This process yielded a total of n = 498 papers for screening.

After removing n = 6 duplicates, n = 492 papers were screened based on their title and abstract. Of these, n = 485 were excluded because they did not meet the inclusion criteria; in particular, n = 480 were excluded for not including the term “complexity” in the title or the abstract (criterion a), and n = 5 did not concern validated NPCSs aimed at assessing pediatric patients’ nursing complexity as defined in this literature review (criterion b). Most retrieved articles were excluded in the initial screening phase because they were not pertinent or investigated complexity from a non-nursing perspective (for example, “health complex conditions” or “medical complexity”).

The seven remaining papers were read in full to assess their eligibility, as their inclusion status was not clear after the initial screening. However, none of these papers met inclusion criterion b. Consequently, they were excluded, so our literature review yielded no results.

As these papers did meet the first inclusion criterion (i.e., the term “complexity” was present in the title or the abstract), they were further analyzed to describe the main characteristics of the reported studies to understand why the research question remained unanswered and to guide future research in this field (Supplementary Table S1). This step was undertaken in accordance with the Cochrane Effective Practice and Organization of Care guide to reporting empty reviews [21].

**Figure 3:**
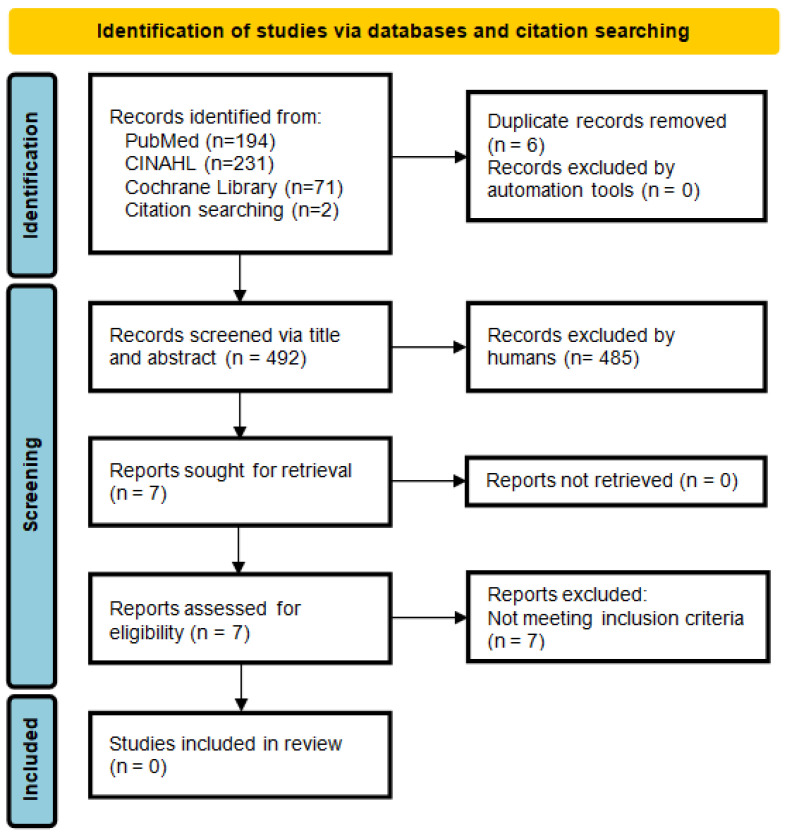
PRISMA flow diagram illustrating the study selection process.

The adapted PRISMA diagram [22] describing the papers’ identification and the selection process is shown in Figure 3.

## 4. Discussion

Our review aimed to provide an overview on validated Nursing Patient Classification Systems (NPCSs) that assess pediatric patients’ nursing complexity, as defined by Richards and Borgling [15] (p. 531), based on the complexity of their nursing care needs. However, our research yielded a critical result: no paper met the defined inclusion criteria.

The seven full-text papers reviewed for eligibility were ultimately excluded either because they did not concern a validated NPCS that assessed pediatric patients’ nursing complexity [23,24,25,26]; they did not concern a validated NPCS that assessed pediatric patients’ nursing complexity as defined in this literature review [27,28,29]; or they focused on concepts that seemed similar but did not align with our specific definition of nursing complexity [15].

A significant finding of this review, in fact, is the conceptual heterogeneity within the literature about the term “complexity”.

Perroca et al. [27] referred to “Patient’s complexity in relation to nursing care”, “Care complexity”, “Nursing complexity”, and “Severity”. De Brito and de Brito Guirardello [28] referred to “Patient’s care complexity”, “Complexity of patients”, “Care complexity”, “Dependency on care” and “Patient care demand”. Connor et al. [29] referred to “Acuity and complexity of patient care”.

No definitions are consistently provided for any of these concepts.

Furthermore, while our review focused on the complexity of the patient, Connor et al. [23,24,25,29] explored complexity related to its impact on nurses (e.g., “Cognitive complexity of pediatric critical care nursing”, “Cognitive workload” or “Cognitive workload complexity”, “Patient acuity–or complexity- in terms of nursing cognitive workload complexity”, “Complexity of pediatric critical care nursing”, and “Complexity of nursing cognitive workload”), shifting the focus from the patient to the nurses.

In addition, while we aimed to provide an overview on validated NPCSs, some authors recommended further testing of the instrument they published [23,24,26,27,29].

Several NPCSs concerning several aspects are available in the literature: the measurement of nursing workload [30,31,32], staffing algorithm development [33], the time nurses spend addressing pediatric patients’ needs or the adequate allocation of healthcare personnel from a quantitative and qualitative point of view [34], and care categories based on the level of dependency from nurses [6,31,34,35,36].

Under the pressure of reduced economic resources and the demand for increasingly complex approaches to clinical management and nursing care, the reorganization of hospitals on the basis of the intensity of care is being widely adopted, in adult settings as well as in pediatric settings.

In such a context, and acknowledging the inherent complexity of hospitalization itself [37], NPCSs could be highly valuable for assessing the nursing complexity of pediatric patients. Accordingly, they could contribute to providing the best possible nursing care to the patients and their families by allocating them to the appropriate setting with the appropriate staff to address their needs [2].

This narrative literature review, while not yielding an answer to our specific research question, brought to light the presence in the literature of several concepts related to complexity that seem similar but are undefined or variably defined. It also identified a critical gap in the field of NPCSs that assess the nursing complexity of pediatric patients, thereby establishing a clear agenda for future research. This prompts some reflections: What do nurses mean when discussing “nursing complexity”? What are the implications for nursing if the term refers to the patient (complexity of the patient’s or of their needs of nursing care) or the nurse (complexity of the care delivered by the nurse or, with an interesting evolution, the cognitive complexity of nursing care)? Given the geographical distribution of the identified papers (Brazil and USA) and the prevalence of the “complexity of care” concept in the Italian literature [2,3,12,13,38], does the scientific community worldwide recognize this terminology, or is it anchored to specific healthcare and/or cultural settings? What concepts should be chosen to measure what, and for which purposes, in order to support the shift to a hospital organization based on the intensity of care?

Although this narrative literature review was conducted using a rigorous method as recommended for high-quality literature reviews [39], it has some limitations. The most critical limitations are the absence of a conceptual framework and the lack of a globally shared definition for “nursing complexity of the patient”. Moreover, the absence of eligible publications, as evidenced by the finding of zero papers, reflects a lack of scholarly focus, discussion, and debate in this field. This vulnerability pertains more to the knowledge base itself rather than to this review.

Other limitations include the potential exclusion of articles published in languages not spoken by the authors, which could lead to the exclusion of potentially interesting articles. Bias could also result from the reviewers’ backgrounds, as their perspectives could have influenced the identification of the studies.

Future studies could expand the knowledge base on this topic by thoroughly exploring the differences among all the published definitions and concepts related to “complexity”. Existing conceptual analysis should be harmonized by taking into account previous findings to achieve a unified and operational definition of “nursing complexity of the patient”. Such a definition would help guide further research and the development of shared systems for classifying patients based on their nursing complexity.

## 5. Conclusions

The results of this review underscore the importance of this issue in the scientific and professional landscape, and we hope it will stimulate further reflection to address the questions raised.

This narrative literature review highlighted a critical gap in the field of validated Nursing Patient Classification Systems (NPCSs) that assess the nursing complexity of pediatric patients. This finding is highly significant as it reveals that the lack of a shared definition of “nursing complexity of the patient” is the primary conceptual barrier to progress in this field. Establishing a unified and actionable definition through the harmonization of existing concepts is the necessary first step to guide future research and the development of shared NPCSs to minimize the variability in assessment practices and support the strategic reorganization of hospital resources.

Once the perspective has been defined—that is, what the term “complexity” specifically refers to—it will be possible to establish a shared terminology and develop appropriate systems for observing, measuring, and analyzing the phenomenon under study.

## Figures and Tables

**Table 1:** Definition of complexity in the Italian context.

Author, Year	Concept	Definition/Dimensions
Galimberti et al. (2012) [12]	Complexity in nursing care	“The whole set of acts that reflect all dimensions of care expressed in terms of intensity, engagement, and quantity of nurses’ work”
Mongardi et al. (2015) [2]	Care complexity	“A set of intervention referring to the different dimensions of nursing care, expressed in terms of the nurse’s commitment and workload”
Mongardi et al. (2015) [2]	Complexity of care	“The interaction of three variables: clinical stability/instability, the patient’s ability to define their needs and the possibility of acting autonomously and effectively”
Rossetti et al. (2016) [13]	Care complexity	“Represented by three concepts, i.e., (a) nursing intensity, described as the quantity of direct and indirect nursing care, (b) nursing workload, described as the necessary resources to nurse patients about the severity of the disease, dependency level and complexity of the cure required and (c) patient acuity, described in terms of severity of the disease and of the intensity of care required as aspects determining care complexity and workload”

## Data Availability

All data related to this study are provided in the manuscript. No new data were created or analyzed in this study.

## Supplementary Materials

The following supporting information can be downloaded at https://www.mdpi.com/article/10.3390/healthcare13222923/s1, Table S1. Summary table of full-text articles excluded (n = 7).

## Abbreviations

The following abbreviations are used in this manuscript:

PCS	Patient Classification System
NPCS	Nursing Patient Classification System
