# Supplementary material for: Nursing Patient Classification Systems to Assess Pediatric Patients’ Nursing Complexity: An Empty Narrative Literature Review
Source: Healthcare (Basel). 2025 Nov 15;13(22):2923. doi: 10.3390/healthcare13222923 (PMC12652381; doi:10.3390/healthcare13222923)
Supplement: Supplementary file 1 [file healthcare-13-02923-s001.zip › healthcare-3788034-supplementary.pdf]

Table S1. Summary table of full-text articles excluded (n = 7)

Authors (Year)	Study Purposes	Study design	Concepts: definitions	Settings	Main outcomes
Connor et al. (2015) [23]	To describe and quantify the cognitive workload and complexity of contemporaneous pediatric critical care nursing.	I Phase: Delphi study with modified Delphi technique.<br>II Phase: not clearly stated.	Cognitive workload : "The intellectual processing of information about patients that drives performance and decision making".<br>Cognitive complexity of pediatric critical care nursing: no definition.<br>Cognitive workload complexity: "The intellectual processing of information about patients that drives decision making and performance".	Freestanding pediatric quaternary hospital.<br>Cardiac Intensive Care Unit.	n=14 domains of nursing representing the cognitive workload of care delivery and five cognitive workload complexity classification groups (I-V) were identified.<br>Classification of patients was 13% as group I or II, 80% as group III or IV, and 7% as group V.
Connor et al. (2019) [24]	To scale and implement a standardized acuity measure of pediatric critical care nursing (CAMEO II).	Delphi study with modified Delphi technique.	Patient acuity in terms of nursing cognitive workload complexity: "The intellectual processing of patient information that drives critical thinking, decision making, and the resulting level of surveillance necessary to meet patient needs".	Quaternary freestanding children hospital.<br>Intensive Care Units (n=4).	The original Domains of Care were confirmed and additional domains were identified.<br>n=18 Domains of Care was confirmed with corresponding care items and cognitive workload scores.<br>Measures of time for certain domains were standardized.<br>The "Standard of Care" list was confirmed.<br>Implementation of the CAMEO II occurred over the course of nine months.

Cnnor et al. (2019) [25]	To describe the acuity and complexity of pediatric critical care nursing and correlate the CAMEO II tool with pediatric physiologic measures.<br>To describe and analyze the reliability and internal validity testing of the CAMEO II.	Descriptive cohort study.	Patient acuity/complexity in terms of nursing cognitive workload complexity: no definition.<br>Complexity of pediatric critical care nursing: no definition.	Quaternary freestanding children hospital. Intensive Care Units (n=4), comprising neonatal and pediatric ones).	Significant correlation between the tool and: the Therapeutic Intervention Scoring System - Children ($p = 0.567$, $P < 0.001$) , the Pediatric Risk of Mortality III ($p = 0.446$, $P < 0.001$), and the Score for neonatal Acute Physiology Perinatal Extension II ($p = 0.359$, $P = 0.013$)
Dini & Guirardello (2013) [26]	To construct a tool for classification of pediatric patients, validate its content, and assess its inter-rater reliability.	Mixed method quantitative study in two steps.<br>I step: descriptive exploratory study using the Delphi technique to construct the instrument and validate its content.<br>II step: correlational study to assess its inter-rater reliability.	Care demand (Demanda de cuidado): no definition.<br>Intensity of nursing need (Necessidade de enfermagem): no definition.<br>Care needs: no definition.	A teaching hospital.	After four stages of use of the Delphi technique, the instrument has acquired its final configuration (11 care demand indicators, each of them comprised one-to-four situations of graded complexity). The reliability levels as optimal, good, and weak were obtained for five, five, and one indicators, respectively.

Perroca et al. (2004) [27]	To analyze the construct validity of Perroca patient classification instrument.	Not clearly stated.	Patient's complexity in relation to nursing care (Complexidade do paciente em relação à atenção de enfermagem): no definition. Care complexity (Complexidade do cuidado): no definition. Nursing complexity (Complexidade assistencial): no definition. Severity (Gravidade): no definition.	School hospital. Medical, Surgical, Obstetrics and Gynecology Clinic, and Intensive Care Units (no Pediatric and Neonatal Intensive Care Units).	The 65.2% of patients were categorized as requiring Minimum Care; the 22.7% as Intermediate Care; the 4.3% as Semi-Intensive Care; and 7.8% as Intensive Care. Among the 13 critical indicators of the instruments, the first two factors explain 75% of the total variation, and the first factor alone represents 66.7% of the total variability. Analysis of Variance shows a clear differentiation between the care categories for Factor 1 and internally within each category for factor 2. A positive correlation was obtained between all the 13 indicators with Pearson ranging from 0.309 to 0.882.
de Brito & de Brito Guirardello (2012) [28]	To evaluate the level of patient`s care complexity.	Descriptive study.	Patient`s care complexity/ Complexity of patients (Complexidade assistencial dos pacientes): no definition. Dependency on care (Dependencia de cuidado): no definition. Care complexity (Complexidade de cuidado): no definition. Patient care demand (Demanda de cuidado): no definition.	Tertiary and quaternary care teaching hospital. Multispecialty unit divided into two stations.	n= 1.080 observations were obtained. There was a predominance of patients requiring high-dependency care, followed by intermediate and semi-intensive care.

Connor et al. (2020) [29]	To development and implement the Inpatient CAMEO© in the pediatric inpatient setting.	Delphi study with modified Delphi technique.	Complexity of nursing cognitive workload: "The intellectual processing of patient information that drives critical thinking, decision-making and the resulting level of surveillance necessary to meet patient needs". Acuity and complexity of patient care: no definition.	Quaternary freestanding children hospital. Pediatric inpatient units (n=11) preterm to 55 years of age).	The existing 18 Domains of Care for the ICU setting were transferable to inpatient nursing. Each of the 18 Domains of Care was confirmed. A number of care items were not applicable to the inpatient setting. Some adjustments were made. Measures of time for certain domains were standardized. A "Standard of Care" list was created. A new baseline score for the Inpatient CAMEO© was developed. Implementation of the Inpatient CAMEO© across the multiple inpatient units occurred over a 12 month period.
